# Are the UK’s vitamin C recommendations evidence-based? A critical comment

**DOI:** 10.1017/S0007114525105941

**Published:** 2026-02-28

**Authors:** Harri Hemilä, Elizabeth Chalker

**Affiliations:** 1 Department of Public Health, University of Helsinki, POB 20, FI-00014 Helsinki, Finland; 2 National Centre for Epidemiology and Population Health, Australian National University, Canberra, ACT 2601, Australia

**Keywords:** CVD, Deficiency diseases, Nutrition policy, Recommended dietary allowances, Respiratory infections, Scurvy, Common cold

## Abstract

There is substantial international variation in recommended vitamin C intake levels. In the USA, the recommendation is 90 mg/d for men and 75 mg/d for women, while in the UK, the current recommendation – established in 1991 – is only 40 mg/d for adults. This UK level was based on the 1953 Sheffield study, which found that 10 mg/d prevents scurvy, with 40 mg/d chosen as the recommended level for yielding somewhat higher plasma levels. In this commentary, we argue that the UK recommendation overlooked key evidence available at the time. Specifically, at least six controlled trials published before 1991 reported benefits from vitamin C supplementation in participants whose baseline vitamin C intake was already 40 mg/d or higher. One randomised controlled trial, published in 1993, found benefits from vitamin C supplementation even at a baseline intake of about 500 mg/d; however, this trial involved ultramarathon runners, and the findings should not be broadly generalised. Nonetheless, such results challenge the assumption that 40 mg/d is universally adequate to maintain full health. We also highlight that the UK recommendations were narrowly focused on preventing dermatological symptoms of scurvy, despite strong evidence – even at the time – that vitamin C deficiency can also cause cardiac dysfunction and greater morbidity due to respiratory infections. We conclude that the current UK vitamin C recommendation should be re-evaluated in light of controlled trial evidence and broader clinical outcomes.

## Graphical abstract

**Figure:**
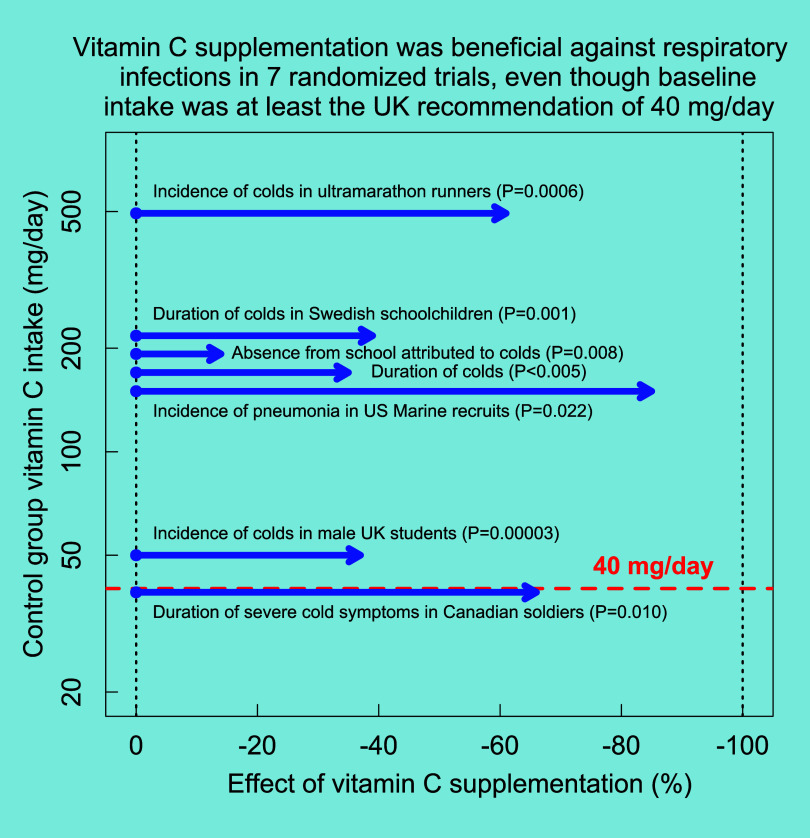

Vitamin C (ascorbic acid) is a key micronutrient that is found naturally in many fruits and vegetables. It is involved in a wide range of biochemical and physiological processes, including the amidation of several peptide hormones and the synthesis of nitric oxide, norepinephrine, carnitine and collagen^(1,2)^. In addition, vitamin C contributes to the modulation of gene expression through epigenetic mechanisms affecting more than a thousand genes^(3)^. Nevertheless, the optimal dosage required to achieve these functions effectively is still not well established.

Vitamin C intake recommendations vary widely between countries. In the USA, the recommended daily amounts are 90 mg/d for men and 75 mg/d for women^(4)^. These levels were chosen to keep vitamin C levels high in certain immune cells with minimal urinary excretion of the vitamin^(4)^. In the UK, the current vitamin C recommendation was established 34 years ago, in 1991, and remains substantially lower than the USA recommendation, set at just 40 mg/d for adults in the UK^(5)^. According to the UK report, ‘10 mg/d is sufficient to protect against scurvy’. The primary focus of the consideration was on dermatological symptoms, stating that ‘the most characteristic clinical signs of diet-induced vitamin C deficiency were failure of hair follicle eruption, the occurrence of petechial haemorrhages spreading to sheet haemorrhage on the limbs, and bleeding gums’^(5)^. The recommendation was set at 40 mg/d – not because this level was considered to have any clinical benefits over 10 mg/d – but because ‘measurable amounts of ascorbic acid begin to circulate in the plasma of most people at an intake of 40 mg/d’^(5)^. The UK report also states that ‘a wide range of other signs and symptoms was reported, but less often’. However, the fact that some symptoms are less common than the dermatological symptoms does not mean that they should be disregarded when considering appropriate vitamin C intake levels.

This commentary focuses on the effects of vitamin C on respiratory infections and argues that strong evidence supporting the benefits of doses higher than 10 and 40 mg/d in some contexts was overlooked in the formulation of the 1991 recommendations. The first reference in the 1991 recommendation is the highly influential Sheffield study on vitamin C deprivation, which led to scurvy in the deprived participants^(6–10)^. In the Sheffield study, the term ‘requirement’ was ‘used to mean the amount of a dietary essential which must be eaten to maintain full health’^(6,7)^. The study found that vitamin C deprivation (∼1 mg/d) nearly doubled the duration of colds compared with those who received 10–70 mg/d vitamin C, and the report stated that ‘such evidence as there is, however, definitely confirms the hypothesis that the absence of vitamin C tended to cause colds to last longer’^(6,10)^; see the Supplement. Thus, the Sheffield study demonstrated that the duration of the common cold is a clinically relevant outcome influenced by vitamin C intake levels. However, that study is not informative about the dose–response relationship between the vitamin C intake level and morbidity due to respiratory infections.

Recently, the data on the impact of vitamin C on scar strength in the Sheffield study were re-analysed^(11)^. The re-analysis argued that the original conclusions were flawed. In the new analysis, vitamin C intake of 10 mg/d was associated with a 42 % weakened scar strength when compared with a vitamin C intake of 80 mg/d. Thus, the scar strength re-analysis challenges the validity of the 10 and 40 mg/d levels.

The purpose of this paper is to analyse controlled trials on vitamin C and respiratory infections, for which there are data about baseline vitamin C intake levels. If 10 mg/d is a ‘sufficient’ intake level ‘to maintain full health’, no significant benefits should be seen from higher doses of vitamin C if the control group intake level is more than 10 mg/d.

## Controlled trials on vitamin C and respiratory infections before 1991

A systematic review published in 1992 identified eighteen placebo-controlled trials, and all of them found that colds were shorter or less severe in groups administered ≥ 1000 mg/d of vitamin C (*P* = 0·000004)^(12)^. The trials were carried out in six countries: the USA, the UK, Canada, Australia, Sweden and Chile. A third of the trials were carried out in children. Although all these trials were published before 1991, none were referred to in the UK recommendations^(5)^.

Another systematic review published in 1997 showed that in four trials with British males, vitamin C administration decreased the incidence of colds on average by 30 % (*P* = 0·000001)^(13)^. In addition, one randomised trial with British males found that 4000 mg/d of vitamin C treatment for the first cold reduced the incidence of recurrent colds by 40 % (*P* = 0·018)^(14)^, and another randomised trial found that 1000 mg/d vitamin C decreased the incidence of ‘chest colds’ in British females by 18 % (*P* = 0·014)^(15)^ (see the Supplement). These six trials are relevant when considering appropriate vitamin C intake levels in the UK; however, none of them were cited in the UK recommendations^(5)^. Chris Bates, the Vice-Chairman of the Panel on Dietary Reference Values Working Group on Vitamins for the 1991 recommendations, was critical of the 1997 meta-analysis, suggesting statistical and selection biases and questioning the reliability of the supporting studies^(16)^. Hemilä addressed the criticism point by point^(17)^. Despite differing views, both authors agreed that more research is needed to better inform vitamin C recommendations^(16,17)^, yet the 1991 UK recommendations still prevail.

Among the more than two dozen trials on vitamin C and the common cold conducted prior to 1991, we identified eight that demonstrated a beneficial effect of vitamin C and provided data on baseline vitamin C intake levels^(18–24)^; see Table 1.

**Table 1.** Vitamin C intake in the control group and the effect of additional vitamin C

Trial ^(ref.)^^*^	Control group vit C (g/d)	Dose of vit C (g/d)	effect of vit C	*P* (1-tail)	Outcome
Trials before 1991					
Miller (1977)^(18)^	0·53	0·5–1	39 %^†^	0·004	Mothers inferred vitamin C administration
Ludvigsson (1977)^(19)^ Pilot study	0·19	1	–39 %	0·0011	Duration of colds
Ludvigsson (1977)^(19)^ Main study	0·17	1	–14 %	0·008	Absence from school because of an upper respiratory tract infection
Carr (1981)^(20)^	0·17	1	–35 %	0·005	Duration of colds
Pitt (1979)^(21)^	0·15	2	–10 %	0·012	Severity of colds
Pitt (1979)^(21)^	0·15	2	–86 %	0·022	Incidence of pneumonia
Baird (1977)^(22)^	0·050	0·08	–37 %	0·00003	Incidence of colds
Sabiston (1974)^(23)^	0·039	1	–59 %	0·027	Incidence of colds
Sabiston (1974)^(23)^	0·039	1	–67 %	0·010	Duration of severe cold symptoms
Glazebrook (1942)^(24)^	0·0125	∼0·2	–23 %	0·017	Incidence of colds treated in sick quarters
Glazebrook (1942)^(24)^	0·0125	∼0·2	–100 %	0·006	Incidence of pneumonia
Glazebrook (1942)^(24)^	0·0125	∼0·2	–50 %	< 0·00001	Days spent in the sick room due to ‘infective conditions’ per schoolboy
Trials after 1991					
Peters (1993)^(29)^ Runners	0·494	0·6	–53 %	0·0006	Incidence of colds

The trials are ordered by the level of vitamin C intake in the control group, except the Peters (1993) trial with runners, which was published after 1991, whereas all the other trials were published before.

**Fig. 1.**
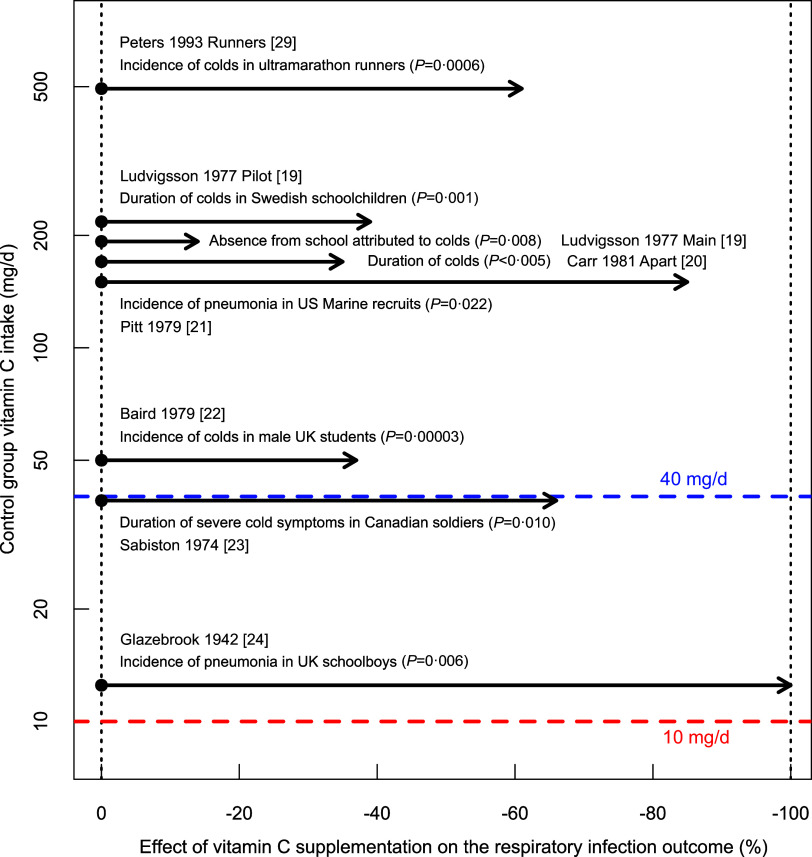
Effect of vitamin C administration on respiratory infection outcomes by baseline vitamin C intake. The vertical axis shows the baseline vitamin C intake in the control groups, with each dot indicating the control group of the trial. Horizontal arrows indicate the effect of vitamin C administration. Data for this figure are presented in Table 1 and described in detail in the Supplement. The assumed ‘sufficient level’ of 10 mg/d and the UK recommendation of 40 mg/d are indicated^(5)^.

* The extraction of data to this table is detailed in the Supplementary file. Pitt (1979), Sabiston (1974) and Glazebrook (1942) reported benefits on more than one outcome, and only one is selected for Fig. 1.

† 39 % (17/44) of the mothers correctly inferred which twin was administered vitamin C, see the Supplement. The Miller (1977) trial is shown in this table since it was a vitamin C and common cold trial with estimated dietary vitamin C intake, though the outcome is not a direct respiratory infection outcome; thus, the trial is not included in Fig. 1.

## Controlled trials on vitamin C and respiratory infections after 1991

The most intensive period of research on vitamin C and the common cold was in the 1970s, with few trials being published after 1991^(25–28)^. Peters *et al*. (1993) found vitamin C to be beneficial for ultramarathon runners and reported baseline vitamin C intake^(29)^. This trial was not available to the 1991 authors, yet it is relevant when considering the current validity of the 34-year-old UK recommendations.

## Effect of vitamin C supplementation by the control group vitamin C level

When the administration of 1000 mg/d of vitamin C has an impact on morbidity due to respiratory infections, the difference between the control and vitamin C groups may be explained either by the particularly high dose in the vitamin C group or by a particularly low intake in the control group. In two therapeutic trials^(30,31)^, there was strong evidence for the former interpretation since there was dose-dependency up to 6000–8000 mg/d in the effect on cold duration^(25–28,32)^.

On the other hand, Glazebrook and Thomson (1942) estimated that baseline vitamin C intake level in UK schoolboys was 10–15 mg/d, yet the low dose of about 200 mg/d of vitamin C decreased the total days spent in the sick room due to infections by 50 %, the incidence of colds treated in the sick room by 23 % and the incidence of pneumonia by 100 % (see Table 1)^(24)^. Baird *et al*. (1979) estimated that the control group received 50 mg/d, yet a low dose of 80 mg/d of vitamin C decreased the incidence of colds in UK male students by 37 %^(22,33)^. Given the low supplement doses in these two trials, we propose that the observed benefits are more likely attributable to the participants’ low dietary vitamin C intake rather than to the doses of the supplementary vitamin C^(13)^.

There are baseline data for vitamin C intake in eight trials, which found significant benefits from vitamin C administration; see Fig. 1. All control groups had a vitamin C intake over 10 mg/d – ‘sufficient’ according to the UK recommendation^(5)^. Peters *et al.* studied ultramarathon runners with baseline vitamin C intakes of about 500 mg/d^(29)^, which is about 50-fold the assumed ‘sufficient’ intake level and over 10-fold the recommendation of 40 mg/d^(5)^; vitamin C administration still reduced common cold incidence significantly. The total number of participants in the 8 trials shown in Fig. 1 is 3367 (median 176), in contrast to just twenty participants in the influential Sheffield study on vitamin C deprivation^(6–10)^. In three trials, the participants were under heavy physical stress^(21,23,29)^, and the findings should not be generalised to sedentary people. Furthermore, along with the randomised trial on ultramarathon runners, Peters (1993) published a parallel trial with sedentary participants and found no benefit from vitamin C supplementation to them, indicating that the level of physical stress can modify the appropriate level of vitamin C intake^(25,26,29,34)^. In the Sheffield study^(6–8)^, participants were sedentary, and similarly, the findings of that trial should not be generalised to people who are physically very active.

The trials in Fig. 1 refute the validity of 10 mg/d as ‘sufficient’ or the ‘requirement’ dose ‘to maintain full health’ over the entire population. Seven of the trials also suggest that 40 mg/d is insufficient for the examined participants since higher doses were associated with significantly better health outcomes.

## Definition of scurvy was not considered

The UK recommendation does not consider the scope of the term ‘scurvy’. The recommendations state: ‘Essential and undisputed roles of vitamin C (ascorbic acid) in man are to prevent scurvy and to aid wound healing’. The Sheffield study is referred to by stating that ‘nearly all individuals who received no more than 1 mg/d for 3–6 months from a specially vitamin C-depleted but otherwise adequate diet developed mild clinical signs of scurvy’^(5)^.

We consider this misleading. In the Sheffield study, 40 % (4/10) of vitamin C-deprived participants suffered from severe health events caused by the deprivation^(6–10)^. Two had an acute cardiac event, the third had an exacerbation of tuberculosis and the fourth had swelling of the knees. Two of these events were triggered by physical stress. In each case, vitamin C administration helped. Thus, nearly half the vitamin C-deprived participants suffered from events that were not just ‘mild clinical signs of scurvy’.

In the 18th century, James Lind described that scurvy causes cardiac dysfunction^(35,36)^, and there were also numerous other reports before the Sheffield study indicating that scurvy causes cardiac dysfunction^(10)^. Therefore, the two cardiac emergencies observed in the Sheffield study were not unexpected events but were consistent with medical literature available prior to the trial. Further evidence on vitamin C and cardiac dysfunction has emerged since the Sheffield study was reported^(37–47)^. The cardiovascular harms of scurvy were not addressed in the UK recommendations^(5)^. Scurvy should not be regarded solely as a dermatological condition characterised by features such as impaired hair follicle function and petechial haemorrhages.

When controlled trials show that low dietary vitamin C intakes increase the risk and severity of respiratory infections in certain people, these effects should also be considered as one integral part of scurvy and should be taken into account when formulating recommendations (Table 1, Fig. 1).

## Individual variation in the effects of vitamin C

The recommendation states that ‘many studies have raised the question of whether vitamin C has beneficial effects on normal human subjects at intakes and tissue levels considerably greater than those needed to prevent or cure scurvy’^(5)^. This is followed by ten references to studies on surrogates, such as immune function, instead of controlled trials on clinically relevant outcomes such as infections.

Thereafter, the recommendation continues that ‘it is impossible to base estimates of requirements directly upon the evidence of these studies, partly because the evidence is conflicting in many areas, partly because those studies which have noted a positive benefit of vitamin C supplements have not defined the minimum dietary requirement needed to achieve it’^(5)^.

This line of reasoning is not well-founded. Individuals have different genes and epigenomes, and their life circumstances vary significantly. Therefore, it is unrealistic to assume a single minimum vitamin C requirement that applies universally across all individuals and lifestyle contexts.

Vitamin E provides a clear example of potential heterogeneity in the effects of vitamins. In the large 6-year ATBC Study involving 29 133 males, the impact of vitamin E supplementation on pneumonia risk was modified by smoking, physical activity, body weight and dietary vitamin C intake – demonstrating that the effect was not uniform across all participants^(25,48,49)^. Similarly, strong evidence of heterogeneity was observed in mortality^(50,51)^. These findings are especially compelling because they come from a single randomised trial that applied consistent intervention and outcome measures. This underscores the plausibility that the effects of vitamin C are also not uniform across the population.

Our Cochrane review (2013) showed that there was very strong evidence that the effect of vitamin C on common cold incidence is different between the general population and people under short-term strenuous physical activity (heterogeneity *P* = 0·000002)^(26)^. In within-trial comparisons, Anderson (1972) found significant heterogeneity in the outcome ‘confined to house’ due to the common cold, such that vitamin C supplementation was beneficial to those who had contact with young children, but not to those without contact (heterogeneity *P* = 0·016), and to participants with frequent colds, but not to those with infrequent colds (heterogeneity *P* = 0·023)^(52,53)^. Sex differences were also seen, with greater benefits in males^(13,14,22,33,54)^. Given this evidence of heterogeneity, there is no justification for ignoring the observed positive findings, arguing that ‘those studies which have noted a positive benefit of vitamin C supplements have not defined the minimum dietary requirement needed to achieve it’.

## Physiological factors potentially affecting vitamin C requirements

As noted above, vitamin C contributes to health and well-being through a wide range of effects on biological functions, including collagen synthesis, gene expression, hormone production, cell differentiation, endothelium and immune activity^(1–3,55–57)^. Because of this biochemical and functional complexity, it is likely that optimal vitamin C intakes are context dependent, varying with individual health status, metabolic demands and environmental exposures.

While physiological studies in animals and humans may not directly determine the optimal vitamin C intake for maintaining full health in humans, they can offer insights into how appropriate intake levels might vary across different life contexts. Strenuous physical activity leads to the elevation of oxidative stress^(58)^, and an electron spin resonance study found that vitamin C administration decreased the levels of free radicals generated during exercise^(59)^. These findings provide a biological rationale for the observed benefits of higher doses of the antioxidant vitamin C in individuals under physical stress^(21,23,25,26,29,34,54)^.

In animals capable of synthesising vitamin C, such as rats and mice, the rate of synthesis is not constant. Physiological stress, such as a cold environment^(60)^, poor nutrition^(61,62)^ and several drugs^(63–66)^, can significantly influence the synthesis rate of the vitamin. While this phenomenon cannot be used to determine recommended intake levels for humans, it suggests that the vitamin C requirements for maintaining full health in humans may vary across different life circumstances and should not be assumed to be fixed.

## Potential limitations in the analysis

One potential limitation of this commentary is our focus on trials reporting positive outcomes. It is possible that unpublished negative studies could lead to publication bias. However, we believe this is an unlikely explanation for our findings.

If vitamin C had no true effect, then by chance alone, only about one in twenty trials would be expected to yield a statistically significant result (*P* < 0·05). Explaining the particularly small *P*-values presented in Table 1 purely by chance would require the existence of thousands of unpublished negative trials that estimated dietary vitamin C intake, which seems implausible.

Moreover, publication bias does not account for within-trial differences such as those observed between sexes^(13,14,22,33,54)^ or between participants with and without regular contact with young children^(52,53)^. Publication bias also fails to explain the highly significant differences found between the general population and individuals under acute physical stress^(25,26,29,34)^.

In this commentary, we focus specifically on the effects of vitamin C on respiratory infections, as several controlled trials have estimated baseline vitamin C intake and thus provide data to examine dose–response relationships. Given this focus, our analysis does not extend to dose–response effects of vitamin C on other health conditions.

Since James Lind’s early work, substantial evidence has accumulated suggesting that low vitamin C intake can contribute to cardiac dysfunction^(35–47)^. However, to our knowledge, no suitable data exist to assess the dose–response relationship for this effect. That said, this does not undermine our analysis. Even if the vitamin C intake required to support cardiac health is lower than the optimal intake for preventing or mitigating respiratory infections, this distinction does not invalidate our conclusions.

On average, adults experience around two colds per year, while children typically have around six. Therefore, the potential impact of vitamin C on respiratory infections carries substantial public health relevance, even if the current UK recommendation of 40 mg/d might be sufficient for other clinical outcomes.

## Conclusions

Several controlled trials published before 1991 found that vitamin C administration can be beneficial for respiratory infection outcomes in some contexts, even though baseline vitamin C intake was substantially over the 10 mg/d ‘sufficient’ level and over the 40 mg/d recommendation. However, these trials were overlooked by the authors of the 1991 UK recommendation^(5)^. A recent re-analysis of the scar strength data of the Sheffield study also challenged the validity of the 10 and 40 mg levels^(11)^.

Low levels of vitamin C intake among the general population have not disappeared. According to one study, 33 % of the surveyed population in the UK had vitamin C intakes of less than 40 mg/d^(67)^, though it is evident that there is substantial variation in the proportions of people with low vitamin C intake from different social groups. However, some segments of the population seem to benefit from vitamin C administration even if their baseline intake is over 40 mg/d (Fig. 1).

Within the current evidence-based medicine context, ideally, the recommendations should be based primarily on controlled trials with clinically relevant outcomes such as those presented in Fig. 1. There is strong evidence that optimal vitamin C intake varies among individuals, and this should be taken into account by investigating differences between population groups.

## Supporting information

Hemilä and Chalker supplementary material Hemilä and Chalker supplementary material

## Supplementary material

For supplementary material/s referred to in this article, please visit https://doi.org/10.1017/S0007114525105941.
