# Supplementary material for: Are the UK’s vitamin C recommendations evidence-based? A critical comment
Source: Br J Nutr. 2025 Dec 22;135(4):381–6. doi: 10.1017/S0007114525105941 (PMC12929015; doi:10.1017/S0007114525105941)
Supplement: Hemilä and Chalker supplementary material [file S0007114525105941sup001.pdf]

Are the UK's Vitamin C Recommendations[1] Evidence-Based? A Critical Comment

Harri Hemilä and Elizabeth Chalker

Harri Hemilä
Department of Public Health, University of Helsinki, Helsinki, FINLAND.
harri.hemila@helsinki.fi
https://orcid.org/0000-0002-4710-307X
https://pubmed.ncbi.nlm.nih.gov/?term=hemila+h+vitamin
https://scholar.google.fi/citations?user=2mkomzUAAAAJ
https://researchportal.helsinki.fi/fi/persons/harri-hemil%C3%A4

Supplement to a paper published in British Journal of Nutrition:
https://doi.org/10.1017/s0007114525105941
https://pubmed.ncbi.nlm.nih.gov/41424103
https://www.cambridge.org/core/journals/british-journal-of-nutrition

Contents **Page**

References to the Sheffield study 2

Sheffield (1953) study: text on the common cold and the summary of the report 3

Statistical issues 7

Table S1: Variations in vitamin C levels in the control and vitamin C groups 12

Table S2: Vitamin C intake in the control group and the effect of additional vitamin C 13

Description of the included trials 14

Note: References are indicated by square brackets []. Reference numbers of the main text are used for papers that are cited in the main text. Papers that are not cited in the main text are listed in this supplement with the "S" at the beginning.

1 The UK vitamin C recommendation (1991) section is available at:
https://doi.org/10.5281/zenodo.13946536
https://wellcomecollection.org/works/pguqct9n/items?canvas=145
https://wellcomecollection.org/works/pguqct9n
https://pubmed.ncbi.nlm.nih.gov/1961974

References to the Sheffield study; numbers refer to the BJN comment:

6. Vitamin C requirement of human adults. *Spec Rep Ser Med Res Counc (GB)*. 1953;280:1-179. https://pubmed.ncbi.nlm.nih.gov/13119061 [see the Summary on p. 5-6 of this Supplement].

7. Vitamin C requirement of human adults; experimental study of vitamin-C deprivation in man. [Summary]. *Lancet*. 1948;1(6510):853-8. https://doi.org/10.1016/S0140-6736(48)90572-8

8. Krebs H. The Sheffield experiment on the vitamin C requirement of human adults. [Summary]. *Proceedings of the Nutrition Society.* 1953;12(3):237-46. https://doi.org/10.1079/PNS19530054

9. Introduction; Historical Account; General Plan; Case Histories; References. In: Vitamin C requirement of human adults. *Spec Rep Ser Med Res Counc (GB)*. 1953;280:1-8,73-88,176-9. https://doi.org/10.5281/zenodo.7661782

10. Hemilä H. Effect of vitamin C deprivation on the duration of colds in the Sheffield study (1953): a statistical analysis. Zenodo 2025. https://doi.org/10.5281/zenodo.14717360

11. Hujoel PP, Hujoel MLA. Vitamin C and scar strength: analysis of a historical trial and implications for collagen-related pathologies. Am J Clin Nutr 2022;115, 8-17. https://doi.org/10.1093/ajcn/nqab262

Concise Account of the Experiment; Summary. In: Vitamin C requirement of human adults. *Spec Rep Ser Med Res Counc (GB)*. 1953;280:1-21,143-4. https://doi.org/10.5281/zenodo.14628064

Diet. In: Vitamin C requirement of human adults. *Spec Rep Ser Med Res Counc (GB)*. 1953;280:56-69. https://doi.org/10.5281/zenodo.14628199

Vitamin C. [Summary]. *BMJ* 1948;2(Nov 6):828-829. https://doi.org/10.1136/bmj.2.4583.828 https://pmc.ncbi.nlm.nih.gov/articles/PMC2091948 https://www.jstor.org/stable/25365390

Vitamin C requirements. [Summary]. *BMJ* 1954;1(Apr 3):806-807. https://doi.org/10.1136/bmj.1.4865.806 https://pmc.ncbi.nlm.nih.gov/articles/PMC2084857

Experimental scurvy. *Lancet*. 1954;263:197-8. https://doi.org/10.1016/S0140-6736(54)91268-4

Waife SO. Man's requirement for vitamin C. *Am J Clin Nutr.* 1954;2(4):273-4. https://doi.org/10.1093/ajcn/2.4.273

Sorby Research Institute. *Wikipedia*. https://en.wikipedia.org/wiki/Sorby_Research_Institute also: https://archives.shef.ac.uk/agents/corporate_entities/143 https://en.wikipedia.org/wiki/Kenneth_Mellanby

Rasmussen, L. (eds) *Human Guinea Pigs, by Kenneth Mellanby: A Reprint with Commentaries*. Philosophy and Medicine, vol. 134; Springer, Cham. [Describes the context for the trial]. https://doi.org/10.1007/978-3-030-37697-0

Krebs H, Martin A. The War Years (1939-1945). In: *Reminiscences and Reflections.* 1981; pp. 119–125. Zenodo 2025. https://doi.org/10.5281/zenodo.14866790

Pemberton J. The BMJ's Nuremberg issue. Nobody died during experiments on vitamin C and vitamin A intakes in Sheffield. *BMJ*. 1997;314:440. https://pubmed.ncbi.nlm.nih.gov/9040399
see comments:
https://pubmed.ncbi.nlm.nih.gov/8973237/ (Weidling)
https://pubmed.ncbi.nlm.nih.gov/9133906/ (Addis)

Pemberton J. Medical experiments carried out in Sheffield on conscientious objectors to military service during the 1939-45 war. *Int J Epidemiol*. 2006;35(3):556-8. https://doi.org/10.1093/ije/dyl020

Commentary: guinea-pigs' private war. *Int J Epidemiol*. 2006;35(3):558-60. https://doi.org/10.1093/ije/dyl031

Pemberton J. Unrecognised scurvy. Signs and requirements. *BMJ*. 2010;340:c590. https://doi.org/10.1136/bmj.c590

Sheffield (1953) study [6,10]: text on the common cold and the summary of the report

INCIDENCE AND DURATION OF COLDS

Throughout the experiment the volunteers recorded the number and duration of the colds they experienced. These data are presented in Table 44 (p. 134). At a glance they seemed to indicate that the average number of colds in the deprived and non-deprived groups did not differ markedly, but that they lasted longer in the deprived group. The material was accordingly submitted to a statistician (C. H. Jowett) for analysis, who summarized his investigation as follows:

"The lengths of all colds were subjected to the transformation $y = 20 \log x$. It was considered that this would make the various distributions on which the statistical tests depended more close to 'normal'. The data analysed consisted of the following:

Colds of members of deprived group before dosing.
Colds of members of supplemented group up to and including July 1945.

Assuming for the moment that time of year had no appreciable effect, the following conclusions emerged from the analysis of variance given in Table 18.

TABLE 18

Analysis of variance of transformed lengths of colds

Source of variation	Sum of squares	Degrees of freedom	Mean square
Difference between groups	431·8	1	431·8 (1)
Differences between individuals within a group ..	1092·1	10	109·2 (2)
Variation of length of cold within an individual ..	1365·8	27	50·6 (3)

The ratio of the mean squares (2)/(3) fell just short of the 5 per cent level of significance, but since it almost attained that level, and moreover for *a priori* reasons, it was considered that differences between individuals did in fact exist; the mean square (1) was accordingly tested against (2), the ratio (1)/(2) being equal to 3·95. This value lies between the 5 per cent level and the 10 per cent level of significance, and hence there is no conclusive evidence of a difference between mean transformed length of colds from supplemented to deprived groups. Such evidence as there is, however, definitely confirms the hypothesis that the absence of vitamin C tended to cause colds to last longer.

The geometric mean length of colds of non-deprived subjects = 3·3 days (4)
The geometric mean length of colds of deprived subjects = 6·4 days (5)

As a further check the seasonal difference in colds was investigated; differences between season lengths were not marked, and the numbers of winter and summer colds did not differ seriously from group to group. It may safely be concluded that the difference between the means (4) and (5) was not a manifestation of the seasonal effect.

In illustration of the seasonal differences, the geometric mean lengths of colds for the 'winter' months and the 'early summer' months are given in Table 19.

TABLE 19

Seasonal incidence and duration of colds

Season	Geometric mean length in group:		No. of colds in group	
	supplemented (days)	deprived (days)	supplemented	deprived
November–February	4·0	7·6	11	8
April–July	3·2	7·2	8	8

Conclusion

The data support the hypothesis that colds of deprived subjects lasted longer, but do not establish it."

In connexion with this result mention should be made of the observation of Glazebrook and Thomson (1942) who studied the incidence and duration of infectious diseases in groups of adolescents living in an institution where the dietary level of vitamin C was very low. The incidence of the common cold and tonsillitis or the average duration of illness due to the common cold was not affected by vitamin C supplements, but the average duration of illness due to tonsillitis was longer in the unsupplemented group.

SUMMARY of the Sheffield study [6, pp.143-144]

The scanned summary is available at the Zenodo repository:
Introduction; Historical Account; Concise Account of the Experiment; **Summary**.
In: Vitamin C requirement of human adults.
Spec Rep Ser Med Res Counc (GB). 1953; 280: 1-21, 143-4.
https://doi.org/10.5281/zenodo.14628064

1. Twenty volunteers were given a diet containing less than 1 mg. vitamin C daily. Three received a vitamin C supplement of 70 mg. daily, seven received 10 mg. and ten had no supplement. No signs of deficiency were observed in those receiving supplements during a period of observation of up to 14 months.
2. All ten volunteers receiving no supplement developed clinical signs of scurvy, though in varying degree. The first changes were enlargement and keratosis of the hair follicles, beginning after 17 weeks of deprivation. Later the enlarged hair follicles became haemorrhagic and formed the characteristic scorbutic spots. Scorbutic gum changes began to appear after 26 weeks of deprivation.
3. Five of the ten deprived volunteers showed a very pronounced exacerbation of the acne present in a mild form at the start of the trial. The exacerbation began after about 22 weeks of deprivation.
4. In one case effusion into both knee joints and ecchymoses of the leg occurred, and two volunteers developed cardiac complications which are described and discussed.
5. A dose of 10 mg. of vitamin C daily given to six of the scorbutic volunteers removed the clinical signs of scurvy in all cases. Within 1 or 2 weeks the scorbutic spots began to fade and within from 7 to 9 weeks the appearance of the skin became normal. The gum lesions responded more slowly, restoration being complete within from 10 to 14 weeks.
6. The volunteers receiving a 70 mg. supplement maintained the initial vitamin C level of the plasma which on an average was 0.55 mg. per 100 ml. In the totally deprived volunteers the vitamin C level of the plasma fell rapidly, reaching about 0.03 mg. per 100 ml. after 37 days and remaining below this value for the rest of the deprivation period. In the volunteers receiving a supplement of 10 mg. the plasma level of vitamin C was of the same order throughout as in the totally deprived group. Thus a difference in the vitamin C intake of 10 mg. daily (enough to prevent and cure clinical scurvy) was not reflected in differences in the plasma level of vitamin C.
7. The volunteers receiving a 70 mg. supplement maintained the initial vitamin C level of the white blood cells which on an average was 16.6 mg. per 100 g. In the totally deprived volunteers the vitamin C level of the white blood cells fell to 1 mg. per 100 g. in 113 days, remaining below this value for the rest of the deprivation period. In the volunteers receiving a supplement of 10 mg. the vitamin C level in the white cells also fell but remained roughly 1 mg. per 100 g. above the value for the deficient group.

8. In the deprived group there was no change in body weight and no increased incidence of infection although colds seemed to last longer. Dark adaptation measurements and audiometry gave no abnormal results. The haemoglobin concentration, red cell count, white cell count and bleeding time showed no significant changes.
9. Conventional tests of so-called capillary strength failed to show correlation with the state of vitamin C depletion.
10. Pains in the back, joints and limbs were reported with increasing frequency by the depleted volunteers as the signs of scurvy developed.
11. No significant variations from normal were observed in the plasma values for protein, urea or phosphatase, or in the albumin-globulin ratio.
12. Experimental wounds were made with the object of studying the process of wound healing. The scars left after such wounds had been excised became haemorrhagic at the height of scurvy. Examination of scar tissue by histological methods and breaking-strain tests gave abnormal findings in the deprived group but not in the two groups receiving a supplement.
13. Saturation tests carried out towards the end of the experiment differentiated between vitamin C intakes above 20 mg. but not between the levels so important in practice of 20 mg. and below. An intake of 10 or 20 mg. (sufficient to cure and prevent scurvy) gave about the same result as an intake of 5 mg. (which is below the safety level).
14. Administration to scorbutic volunteers of a tyrosine supplement, which increased the normal daily tyrosine intake from about 5-6 g. to 25 g., did not lead to an increase in urinary excretion of tyrosine and its derivatives as measured by the phenol test of Folin and Ciocalteu.
15. The changes in the capillaries round the hair follicles were examined by capillaroscopy and are described in detail.
16. The urine of deprived volunteers when the true vitamin C content must have been near zero was used for testing the specificity of vitamin C estimations. Direct titration with 2:6-dichlorophenolindophenol gave values between 13 and 48 mg. It is pointed out that the quantity of material behaving like vitamin C in this method is likely to vary with the diet. The dinitrophenylhydrazine method of Roe and Kuether and the dichlorophenolindophenol-formaldehyde method are more specific, but interfering substances simulating vitamin C were still not completely eliminated.
17. The vitamin C requirements of human adults are considered in the light of the results of the present trial. The fact that a supplement of 10 mg. daily cured clinical scurvy in all six cases examined, together with the observation that 10 mg. daily protected seven volunteers for periods of up to 424 days, are taken to indicate that in the group under test the minimum protective dose of vitamin C, measured by the presence or absence of the signs of scurvy, was in the region of 10 mg. daily. In order to arrive at a figure for a daily allowance which covers individual variations and includes a safety margin, it is suggested that the minimum protective dose of 10 mg. be trebled. An allowance of 30 mg. daily is in accordance with the recommendation by the League of Nations Health Organisation Technical Commission on Nutrition made in 1938.

Statistical issues

mid-P

In 2×2 tables, when there are only a few cases in one group, the mid-P value is the most appropriate method to calculate the P values for the differences in the treatment groups [S1-S4]. Table 7 from [S1] illustrates the rationalization, see below. The mid-P was used when comparing groups with small numbers of cases.

S1. Hemilä H. Do vitamins C and E affect respiratory infections?
University of Helsinki, Helsinki, Finland 2006: p.21 (Table 7) [see below].
https://hdl.handle.net/10138/20335
https://doi.org/10.5281/zenodo.6395595

S2. Lancaster HO. Significance tests in discrete distributions. J Am Stat Assoc. 1961;56:223-34.
https://doi.org/10.2307/2282247
https://www.jstor.org/stable/2282247

S3. Berry G, Armitage P. Mid-P confidence intervals. Statistician. 1995;44:417-23.
https://doi.org/10.2307/2348891
https://www.jstor.org/stable/2348891

S4. Lydersen S, Fagerland MW, Laake P. Recommended tests for association in 2 x 2 tables.
Stat Med. 2009;28:1159–75.
https://doi.org/10.1002/sim.3531

1-tailed P

P(1-tail)-values are shown in this analysis for the difference between the high and low vitamin C groups, since the main question is whether a higher level of vitamin C intake decreases the incidence or severity of respiratory infections or not, and this question is unidirectional.
If the P(1-tail) is greater than about 0.97, then there is a reason to assume harm in the treatment group of the particular trial. If a reader prefers the 2-tailed P-value, it is twice the 1-tailed P-values for P<0.5.
For the heterogeneity test P-values we use the 2-tailed test since the direction of the assumed effect is not as clear.

Relative scale

In this commentary, we are using the relative scale, i.e., percentage effects, instead of difference in days in the duration of colds. It has been shown that the relative scale much better captures the effect of treatments on many continuous outcomes such as the duration of colds [S5-S9].

S5. Friedrich JO, Adhikari NK, Beyene J. Ratio of means for analyzing continuous outcomes in meta-analysis performed as well as mean difference methods. J Clin Epidemiol. 2011;64:556–64.
https://doi.org/10.1016/j.jclinepi.2010.09.016

S6. Hemilä H. Many continuous variables such as the duration of the common cold should be analyzed using the relative scale. J Clin Epidemiol. 2016;78:128-9.
https://doi.org/10.1016/j.jclinepi.2016.03.020
https://hdl.handle.net/10138/173096

S7. Hemilä H. Duration of the common cold and similar continuous outcomes should be analyzed on the relative scale: a case study of two zinc lozenge trials. BMC Med Res Methodol. 2017;17:82.
https://doi.org/10.1186/s12874-017-0356-y
https://www.ncbi.nlm.nih.gov/pmc/articles/PMC5427521

S8. Hemilä H, Chalker E, Tukiainen J. Quantile treatment effect of zinc lozenges on common cold duration: a novel approach to analyze the effect of treatment on illness duration.
Front Pharmacol. 2022;13:817522.
https://doi.org/10.3389/fphar.2022.817522
https://www.ncbi.nlm.nih.gov/pmc/articles/PMC8844493
and
https://doi.org/10.3389/fphar.2024.1335784
https://pmc.ncbi.nlm.nih.gov/articles/PMC11035776

S9. Hemilä H, Pirinen M. Estimating quantile treatment effect on the original scale of the outcome variable: a case study of common cold treatments. *Trials*. 2025;26:541.
https://doi.org/10.1186/s13063-025-09265-z
https://pmc.ncbi.nlm.nih.gov/articles/PMC12645726

Multiple comparison issues

Some of the trial reports published several P-values.

In Table V, **Ludvigsson (1977)** [19] published 12 P-values for the "Pilot trial" and 12 P-values for the "Main trial", i.e., 24 P-values in one single table. The set of 12 outcomes were composed of 3 different common cold definitions and 4 different outcomes (free from symptoms, incidence, duration, and the product between incidence and duration).

When we expect – purely by chance – on average one "significant" ($P < 0.05$) P-value per 20 independent measurements, the two significant P-values in Ludvigsson's Table V might be explained just by chance.

On the other hand, the effect on [19]:
"Duration of absence from school" per episode $Z = 2.42$ corresponds to P(1-tail) = 0.008 [Main trial]
"Duration of upper respiratory tract infection" per episode $Z = 3.05$ corresponds to P(1-tail) = 0.001 [Pilot trial]

These are much lower than the (arbitrary) limit of $P = 0.05$.
$P = 0.008$ would require about 125 independent P-value calculations to explain the finding just by chance.
$P = 0.001$ would require about 1000 independent P-value calculations to explain the finding just by chance.
Therefore, it is unlikely that the P-values reported by Ludvigsson are just examples of random variation.

Furthermore, of the 2×12 P-values for the two trials, 2×3 were for the outcome "totally free from symptoms", 2×3 were for common cold incidence, 2×3 were for the duration of episodes, and 2×3 were for the product between incidence and duration. Both the small P-values were for the duration of episodes and this is consistent with the pooled results in our Cochrane review (2013) [26]. None of the 2×3 measures on incidence of colds found a significant benefit, which is also consistent with the pooled results in our Cochrane review (2013) [26]. Thus, although Ludvigsson (1977) calculated several P-values [19], the 2 positive findings corresponded to particularly small P-values in the group of 6 P-values, and they were consistent with the other trials reporting on vitamin C for common cold duration.

Several P-values were also calculated by **Carr** (1981) [20], **Glazebrook** (1942) [24], **Peters** (1993) [29], **Pitt** (1979) [21], and **Sabiston** (1974) [23]. The reported findings in these trials are also consistent with the findings of other trials in the medical literature and so are not unexpected [12,13,25-27,34,53].

Calculation of the P-value for the Tyrrell (1977) recurrent colds in males, and Elwood (1976) chest colds in women.

Tyrrell (1977) [14] reported in Table 3:
https://doi.org/10.1136/jech.31.3.189
https://pmc.ncbi.nlm.nih.gov/articles/pmc479021

```
No. male participants
Placebo     392
Vitamin C   351

No. male participants with 1 cold
Placebo     98
Vitamin C   101

No. male participants with ≥2 colds
Placebo     43
Vitamin C   23
```

Calculation with R:

Limiting to those with colds:

```
> ormidp.test(23, 43, 101, 98, or = 1)
  one.sided two.sided
1    0.0127    0.0255
```

Including all randomized participants:

```
> riskratio.small(matrix(c(349,328, 43,  23 ),nrow=2))
$data
          Outcome
Predictor  Disease1 Disease2 Total
  Exposed1      349       43   392
  Exposed2      328       23   351
  Total         677       66   743

$measure
          risk ratio with 95% C.I.
Predictor  estimate lower upper
  Exposed1    1.000    NA    NA
  Exposed2    0.585  0.36 0.951

$p.value
          two-sided
Predictor  midp.exact fisher.exact chi.square
  Exposed1         NA           NA         NA
  Exposed2     0.0349       0.0387     0.0346

$correction
[1] FALSE

attr(,"method")
[1] "small sample-adjusted UMLE & normal approx (Wald) CI"
> (P_1tail=0.0349/2)
[1] 0.0174
```

Elwood (1976) [15] reported in Table III:
https://doi.org/10.1136/jech.30.3.193
https://www.ncbi.nlm.nih.gov/pmc/articles/pmc478963

```
No. participants
Placebo     349
Vitamin C   339

Placebo     266 "Chest colds"
Vitamin C   211 "Chest colds"
```

Calculation with R:

```
> poisson.test(c(211, 266), c(339, 349))
      Comparison of Poisson rates
data:  c(211, 266) time base: c(339, 349)
count1 = 211, expected count1 = 235.03, p-value = 0.02798
alternative hypothesis: true rate ratio is not equal to 1
95 percent confidence interval:   0.6783813 0.9820592
sample estimates:  rate ratio  0.8166323

> (P_1tail= 0.02798/2)
[1] 0.01399
```

Table S1: Variations in vitamin C levels in the control and vitamin C groups

Trial [ref.]	Control group vitamin C (g/day)			Vitamin C group vitamin C (g/day)	
	**Diet**	**Supplement**	**Total**	**Supplement**	**Total**
Miller (1977) [18]	0.48 [a)]	0.050	0.53	0.5 –1.0	0.98 – 1.48
Peters (1993) Runners [29]	0.285 [b)]	0.209 [c)]	0.494	0.6	1.139
Ludvigsson (1977) Pilot [19]	0.16 [a)]	0.03	0.19	1	1.16
Ludvigsson (1977) Main [19]	0.16 [a)]	0.01	0.17	1	1.16
Carr (1981) Apart [20]	0.1 [d)]	0.07	0.17	1	1.1
Pitt (1979) [21]	0.15 [a)]		0.15	2	2.15
Baird (1977) [22]	0.050		0.050	0.080	0.130
Sabiston (1974) [23]	0.039		0.039	1	1.04
Glazebrook (1942) [24]	0.0125		0.0125	0.2	0.21

[a)] Dietary intake was estimated from reported blood or urine vitamin C levels; see “Description of the included trials”.

[b)] Supplementation by one’s own initiative: Peters (1993) [29].

[c)] Calculated from the published Total and Supplement doses.

[d)] Dietary intake in the Carr (1981) [20] trial is assumed. The 70 mg/day vitamin C supplement in the placebo group already is nearly twice the UK recommendation of 40 mg/day. Therefore, the assumption of dietary intake of 0.10 or 0.05 does not influence the fact that the control group vitamin C level was substantially higher than the UK recommendation.

Table S2: Vitamin C intake in the control group and the effect of additional vitamin C

Trial	Control group vit C<br>vit C (g/day)	Dose of vit C<br>(g/day)	Effect of vit C	P(1-tail) [a]	Outcome
Miller (1977)	0.53	0.5-1	39% [b]	0.004	Mothers could infer vitamin C administration
Peters (1993) Runners	0.494	0.6	-53%	0.0006	Incidence of colds
Ludvigsson (1977) Pilot study	0.19	1	-39%	0.0011	Upper respiratory tract infection
Ludvigsson (1977) Main study	0.17	1	-14%	0.008	Absence from school because of upper respiratory tract infection
Carr (1981)	0.17	1	-35%	0.005	Duration of colds
Pitt (1979)	0.15	2	-10%	0.012	Severity of colds
Pitt (1979)	0.15	2	-86%	0.022	Incidence of pneumonia
Baird (1977)	0.050	0.08	-37%	0.00003	Incidence of colds
Sabiston (1974)	0.039	1	-59%	0.027	Incidence of colds
Sabiston (1974)	0.039	1	-67%	0.010	Duration of severe cold symptoms
Glazebrook (1942)	0.0125	~0.2	-23%	0.017	Incidence of colds treated in Sick Quarters
Glazebrook (1942)	0.0125	~0.2	-100%	0.006	Incidence of pneumonia
Glazebrook (1942)	0.0125	~0.2	-50%	<0.00001	Days spent in the sick room due to ‘infective conditions’ per schoolboy

[a] The P-value shown in this table are marked in bold in the following section which summarizes the included trials.

[b] 39% (17/44) of the mothers could correctly infer which twin was administered vitamin C, see the description of the Miller (1977) trial in the section “Methods of the included trials”. This study is shown in Tables S1 and S2 since it is a vitamin C and common cold trial. However, although the small P-value indicates physiological effects of vitamin C administration, the outcome is not a direct respiratory infection outcome and therefore the trial is not included in Figure 1.

Description of the included trials

**Baird (1979) [22]**	https://doi.org/10.1093/ajcn/32.8.1686<br>https://pubmed.ncbi.nlm.nih.gov/463806
Methods	Placebo-controlled RCT. Duration 10 weeks.
Participants	UK."All were either 6th form pupils at a local comprehensive school or university students" (p.1686).<br>Our data are restricted to males of the trial.<br>Placebo 61 males; vitamin C 133 males.
Intervention	0.08 g/d vitamin C.<br>"The yellow (control) group received daily a synthetic orange juice drink containing no flavonoid material and no AA [ascorbic acid; N=61]; the blue group received the synthetic orange juice with 80 mg of AA added [N= 71]; the red group received natural orange juice containing 80 mg of AA [N= 62]" (p.1687).
Outcomes	Incidence and duration of colds.<br>Days on which they were absent from their studies because of illness.
Baseline vit C intake	**50 mg/day**
Source of dose	"In the current study… the estimated daily intake from dietary sources was 50 mg" (p.1686).
Notes	There was a statistically highly significant interaction between sex and the effect of vitamin C [33]. There are also other trials in which vitamin C was more effective in males [13,14,54].

**Risk of bias**	**Authors' judgment**	**Support for judgment**
Random sequence generation (selection bias)	Low risk	"randomly allocated to three color-coded groups, yellow, blue , and red" (p.1687).
Allocation concealment (selection bias)	Low risk	"allocated to three color-coded groups, yellow, blue, and red; there was no deliberate equalization of age/sex distribution between the three groups. Color coding was necessary to facilitate the daily distribution and consumption of drinks" (p.1687).
Baseline balance	Low risk	No report, but moderately large randomized groups usually have quite balanced groups.
Blinding of participants and personnel (performance bias)	Low risk	"Participants were not informed of the nature of their drinks until the conclusion of the experiment" (p.1687).<br>"the distribution points for the three groups were spatially removed from each other, thus reducing the possibility of "drink comparisons" between the groups" (p.1687).
Blinding of outcome assessment (detection bias)	Low risk	"Participants were required to complete daily a record card by indicating whether they had been well, or whether they had experienced one or more of a number of symptoms usually associated with the common cold" … "Participants were not informed of the nature of their drinks until the conclusion of the experiment" (p.1687).<br>"Record cards were collected at the end of each month and transferred in toto and without grouping, to the statisticians, who were not aware of the separate group supplements, but only a participants group color" (p.1687).
Incomplete outcome data (attrition bias)	Low risk	"one (red group) with evidence of chronic bronchitis was eliminated after examination of the record cards by an independent clinician and two (both in yellow groups) were eliminated because they took daily mega supplements of vitamins" (p.1687). This is 3 out of 353 for both sexes, i.e. 1%.
Selective reporting (reporting bias)	Low risk	Incidence and duration of colds were reported.<br>Days on which they were absent from their studies because of illness were also reported.

Vitamin C and placebo indistinguishable?	Low risk	"the distribution points for the three groups were spatially removed from each other, thus reducing the possibility of "drink comparisons" between the groups" (p.1687).

	**Placebo**	**Vitamin C**
Participants	61	133
Colds	135	184
Difference		-37%
		**P=0.00003**

```
poisson.test(c(184, 135), c(133, 61))

                                Comparison of Poisson rates

data:  c(184, 135) time base: c(133, 61)
count1 = 184, expected count1 = 218.7, p-value = 4.943e-05
alternative hypothesis: true rate ratio is not equal to 1
95 percent confidence interval:
 0.4978929 0.7864937
sample estimates:
rate ratio
 0.6251184

> (P_1tail=0.0000494/2)
[1] 2.47e-05
```

**Carr (1981) Apart [20]**	https://doi.org/10.1017/s0001566000006450<br>https://pubmed.ncbi.nlm.nih.gov/7048833
Methods	Double-blind RCT. Regular supplementation trial. Duration 100 days<br>Identical twins: 1 group living together and the other living apart<br>This study includes those living apart.
Participants	Australian males and females age range 14 to 64 years (mean 25 years). Data were analysed for 38 male and 57 female pairs of twins in total (36 pairs under 18 years, 34 pairs aged 18 to 30, 25 pairs aged 30+).<br><br>This study is restricted to 51 twin pairs living apart.
Intervention	1 g/d vitamin C.
Outcomes	Incidence, duration and severity of colds.
Baseline vit C intake	**170 mg/day**<br>Both groups received a multi-vitamin tablet containing 70 mg/d vitamin C. This was to ensure that any observed treatment effect could reasonably be attributed to the pharmacologic dose of vitamin C and not to alleviation of dietary deficiency.
Source of dose	We assume dietary vitamin C intake of 100 mg/day. Together with the supplement the control group intake is 170 mg/day.<br>Our analysis is not sensitive to the assumption of dietary intake, since the placebo group vitamin C intake already is nearly double the UK vitamin C recommendation. Thus, assuming eg 50 mg/day dietary intake would not materially change our analysis.
Notes	Carr (1981) found that vitamin C had a beneficial effect on the duration of colds for twins living separately, but not for twins living together. This subgroup difference might be explained by swapping of tablets by twins living together, which was not possible for twins living separately. Furthermore, the duration of colds among twins living together (5.4 days in vitamin C and placebo groups) was in the middle of the duration of colds among the vitamin C group (4.9 days) and placebo group (7.5 days) of twins living apart.<br><br>In trials with children, Miller found an increase in vitamin C levels in the urine of boys of the placebo (sic) groups [18], which also indicates tablet swapping between the children on vitamin C and placebo.<br><br>For these reasons we consider that the results for the twins living apart are more valid than the results for the twins living together.<br><br>Funding: Roche Products, supplied the tablets and gave financial support to cover postage costs

**Risk of bias**	**Authors' judgment**	**Support for judgment**
Random sequence generation (selection bias)	Low risk	"One twin of a pair was assigned at random" (p.250)
Allocation concealment (selection bias)	Low risk	"The experiment was 'double-blind' in that neither the subjects nor the experimenters involved with the subjects or with the analysis of the results knew which group was which until the experiment and the analysis were completed" (p.250).
Baseline balance	Low risk	Twins
Blinding of participants and personnel (performance bias)	Low risk	"The experiment was 'double-blind' in that neither the subjects nor the experimenters involved with the subjects or with the analysis of the results knew which group was which until the experiment and the analysis were completed" (p.250).
Blinding of outcome assessment (detection bias)	Low risk	"The experiment was 'double-blind' in that neither the subjects nor the experimenters involved with the subjects or with the analysis of the results knew which group was which until the experiment and the analysis were completed" (p.250).

Incomplete outcome data (attrition bias)	Low risk	“Of the 125 pairs of twins who began the trial, we have analyzed cold data for 95 pairs” (p.250)<br>Whole pairs were excluded, so that exclusion cannot cause systematic differences between the groups.
Selective reporting (reporting bias)	Low risk	Incidence of colds, duration of colds and severity of colds reported.
Vitamin C and placebo indistinguishable?	Low risk	“matching of the active and placebo tablets was checked for both appearance and taste” (p.250).

	**Placebo**	**Vitamin C**
Participants	44	44
Duration of colds, days	7.50	4.86
Difference		-35%<br>**P = 0.005**<br>Table 2 [20]: P(2-tail) < 0.01

**Glazebrook (1942) [24]**	https://doi.org/10.1017/s0022172400012596<br>https://pmc.ncbi.nlm.nih.gov/articles/PMC2199803
Methods	Non-random allocation by divisions of the boarding school.<br>Quasi-placebo controlled trial with allocation in administrative units.<br>This table shows a brief summary, but for further details of methods, see extracts of the report below on pp.19-21.
Participants	Schoolboys in a boarding school before World War II.<br>335 vitamin C 335; 1100 control.<br>"In a large training school under our observation there were some 1500 youths aged 15-20 years. For the most part they were drawn from the lower wage-earning classes, and a large proportion came from Scotland and the North Midlands, where economic conditions are probably below the average for the country. It is a reasonable assumption that the previous dietary of the recruits had been somewhat deficient in vitamin C judged by the standards already quoted (p.4).
Intervention	~0.2 g/d vitamin C, ranging from 0.05 to 0.3 g/d in different periods.
Outcomes	Incidence of colds and the proportion treated in the Sick Quarters and the duration of treatment.<br>Incidence of "tonsillitis" and the proportion treated in the Sick Quarters and the duration of treatment. Authors described: "The term 'tonsillitis' is used here to be an index of haemolytic streptococcal disease of the nose and throat, and covers all such terms as 'tonsillitis', 'sore throat', 'otitis media', 'pharyngitis' and 'cervical adenitis', as nearly all these cases are of haemolytic streptococcal origin. Throat swabs were taken of large numbers of cases of tonsillitis to determine that the haemolytic streptococcus was the causative organism" (p.12).<br>Incidence of pneumonia and rheumatic fever. [pneumonia] "These cases were-subjected to special investigations by us (X-rays, etc.) to establish certain criteria for the diagnosis" (p.16).<br>Days spent in the sick room due to 'infective conditions' per boy
Baseline vit C intake	**10-15 mg/day**
Source of dose	"The total intake of vitamin C varied from about 10 to 15 mg per student per day" (p.5).
Notes	The trial is old.<br>However, pure vitamin C was already available and was used.<br>The method for diagnosing pneumonia is relevant also from the current viewpoint.<br>Similarly outcomes for common cold and tonsillitis are relevant also in the current viewpoint, as well as the count of days spent in the sick room due to infections.<br><br>On the following pages, there are extracts of the trial report relevant to consideration of the methodology.

Risk of bias	Authors' judgment	Support for judgment
Random sequence generation (selection bias)	Low risk	"The establishment was divided into seven groups or divisions for administrative purposes. The youths of one division worked as a unit, and occupied certain tables in the dining hall. To some extent each division occupied particular dormitories but this separation was not absolute, and there was a fair amount of mixing of divisions in the sleeping quarters. Sleeping and feeding conditions were, of course, the same for all divisions" (p.12).<br>"Careful records had been kept of the incidence of all infections for 1½ years before the observations described here were begun. In the preceding year there had been an epidemic of tonsillitis, which had affected all the divisions uniformly, so that they could not be regarded as separate units within the larger populations" (p.12).<br><br>The above suggests that exposure to infectious agents was reasonably uniform over the study population.

Allocation concealment (selection bias)	Low risk	"Pure ascorbic acid powder was added to… the morning cocoa, and an evening glass of milk. The mixing was done in bulk in the kitchens before issue. The powder dissolved quickly and easily, and did not alter the appearance or taste of the vehicle" (p.7).
Baseline balance	Low risk	All were at the same boarding school and all had the same foods.
Blinding of participants and personnel (performance bias)	Low risk	"Pure ascorbic acid powder was added to… the morning cocoa, and an evening glass of milk. The mixing was done in bulk in the kitchens before issue. The powder dissolved quickly and easily, and did not alter the appearance or taste of the vehicle" (p.7).
Blinding of outcome assessment (detection bias)	Low risk	"When a youth felt ill he was admitted to Sick Quarters unless his complaint was very mild… The admission to and discharge from the hospital was not under our control" (p.13).<br>[As to pneumonia:] "These cases were subjected to special investigations by us (X-rays, etc.) to establish certain criteria for the diagnosis" (p.16).
Incomplete outcome data (attrition bias)	Low risk	Closed boarding school.<br>Concluding from the trial report, there were no drop-outs.
Selective reporting (reporting bias)	Low risk	Reporting was extensive and several comparisons with P>0.05 were reported.
Vitamin C and placebo indistinguishable?	Low risk	"Pure ascorbic acid powder was added to… the morning cocoa, and an evening glass of milk. The mixing was done in bulk in the kitchens before issue. The powder dissolved quickly and easily, and did not alter the appearance or taste of the vehicle"

Findings in the Glazebrook and Thomson (1942) trial [24]

Variable	Placebo	Vitamin C	Difference	P [a)]
Participants	1100	335		
Vitamin C dose (mg/day)	10-15	50-300		
Common colds	286	72	-17%	0.047
Colds treated in the Sick Quarters	253	59	-23% [b)]	**0.017**
Days in hospital	6.4	6.32		
Tonsillitis [c)]	94	29	0%	
Admitted to hospital	83	18	-30% [b)]	0.086
Days in hospital, mean	16.7	10.1	-40%	0.0026
Days in hospital, SD	11.86	6.96		
Days in hospital per infected [d)]	14.7	6.3	-57%	0.0021 [d)]
Pneumonia	17	0	-100%	**0.006**
Rheumatic fever [e)]	16	0	-100%	0.007
Days spent in the sick room due to 'infective conditions' per boy, mean	4.98	2.5	-50%	**<0.00001** [f)]

[a)] P(1-tail) is shown. For the 2×2 tables, the mid-P is calculated. See the section "statistical issues" in this supplement.

[b)] Difference and P-value calculated from the population of all participants.

[c)] Authors stated: "The term 'tonsillitis' is used here to be an index of haemolytic streptococcal disease of the nose and throat, and covers all such terms as 'tonsillitis', 'sore throat', 'otitis media', 'pharyngitis' and 'cervical adenitis', as nearly all these cases are of haemolytic streptococcal origin. Throat swabs were taken of large numbers of cases of tonsillitis to determine that the haemolytic streptococcus was the causative organism" (p.12).

[d)] Days in hospital per infected schoolboy.
In the placebo group: 83×16.7/94 = 14.7 days.
In the vitamin C group: 18×10.1/29 = 6.3 days.
Combination of the tonsillitis P-values using Fisher's method:
https://en.wikipedia.org/wiki/Fisher%27s_method

```
> (1- pchisq(-2*(log(0.086) + log(0.0026)),4))
[1] 0.0021
```

[e)] Glazebrook and Thomson do not specify the criteria they used to diagnose rheumatic fever. Refer to the comments on page 19 for more details.

[f)] The SD values were not reported for "days spent in the sick room". In most vitamin C common cold trials the SD for the duration has been on average 70% of the mean duration of colds [26]. Here we use a conservative imputation of SD being 100% of the mean duration, see below for the calculation.

Calculating the P-value for “Days in hospital due to tonsillitis” and “Days spent in the sick room”, using the Ratio of Means approach, see Appendix 4.

```
                           Study  Ne    Me   Se   Nc  Mc Sc ROM LnROM   SDe     SDc SElnROM     z       p
1           Friedrich (test)   9 213.0 67.0   10 177 40 1.2  0.19 0.011 0.00511   0.127   1.5 9.3e-01
2 Glazebrook_SickTonsillitis  18  10.1  7.0   83  17 12 0.6 -0.50 0.026 0.00608   0.180  -2.8 2.6e-03
3         Glazebrook_SickRoom 335   2.5  2.5 1100   5  5 0.5 -0.69 0.003 0.00091   0.062 -11.0 1.2e-28
```

```
> riskratio.small(GTcolds)
$data
         Outcome
Predictor  Disease1 Disease2 Total
  Exposed1      814      286  1100
  Exposed2      263       72   335
  Total        1077      358  1435
$p.value
         two-sided
Predictor  midp.exact fisher.exact chi.square
  Exposed2   0.093467     0.097693   0.095063
> (P_1tail=0.09347/2)
[1] 0.0467
```

```
> riskratio.small(GTColdsSick)
$data
         Outcome
Predictor  Disease1 Disease2 Total
  Exposed1      847      253  1100
  Exposed2      276       59   335
  Total        1123      312  1435
$measure
         risk ratio with 95% C.I.
Predictor  estimate   lower   upper
  Exposed2  0.76342 0.59123 0.98575
$p.value
         two-sided
Predictor  midp.exact fisher.exact chi.square
  Exposed2    0.03436     0.040917   0.036332
> (P_1tail=0.03436/2)
[1] 0.0171
```

```
> riskratio.small(GTpneumonia)
$data
         Outcome
Predictor  Disease1 Disease2 Total
  Exposed1     1083       17  1100
  Exposed2      335        0   335
  Total        1418       17  1435
$p.value
         two-sided
Predictor  midp.exact fisher.exact chi.square
  Exposed2   0.010581     0.017995   0.022082
> (P_1tail=0.01058/2)
[1] 0.00529
```

```
> riskratio.small(GTrheumfever)
$data
         Outcome
Predictor  Disease1 Disease2 Total
  Exposed1     1084       16  1100
  Exposed2      335        0   335
  Total        1419       16  1435
$p.value
         two-sided
Predictor  midp.exact fisher.exact chi.square
  Exposed2   0.013852     0.031415    0.02643
> (P_1tail=0.01385/2)
[1] 0.00692
```

Allocation to treatment groups

In the Glazebrook and Thomson trial [24], allocation to treatment groups was carried out by institute ‘divisions’ and not on the basis of individual boys. Therefore, in the Cochrane review on vitamin C and pneumonia (2013) the trial was also analysed using the ’division’ as the unit of observation [26].

Distribution of pneumonia cases in the five control divisions was 5, 3, 2, 4 and 3 (mean 3.40 cases per division) and in the two vitamin C divisions it was 0 and 0. Assuming that the mean of the control divisions was a suitable estimate for the Poisson distribution mean provided basis for the statistical analysis.

The size of the individual divisions was not stated in the paper but the two vitamin C divisions had on average 167 boys (335/2) and the five control divisions 220 boys (1100/5), thus the size of the vitamin C divisions was 0.761 times the size of the control divisions. The mean incidence was adjusted by this ratio, such that we expected 2.59 pneumonia cases per vitamin C division, assuming the same average incidence as for the control divisions. With this Poisson mean, the probability that there were no cases of pneumonia in two separate vitamin C divisions was calculated as having a P-value of 0.0056. Accordingly, using a ’division’ as the unit of observation does not change the conclusions.

```
> (Control <- mean(c(5,3,2,4,3)))
[1] 3.4
> (RatioBoys <- (335/2)/(1100/5))
[1] 0.761
> (lambda <- Control*RatioBoys)
[1] 2.59
> dpois(0, lambda)^2
[1] 0.0056
```

Diagnosis of rheumatic fever

Glazebrook and Thomson noted regarding pneumonia and rheumatic fever that “These cases were-subjected to special investigations by us (X-rays, etc.) to establish certain criteria for the diagnosis”.

While the diagnosis of pneumonia in the late 1930s appears consistent with the modern understanding of the condition, the same cannot confidently be said for rheumatic fever. The Jones criteria, which currently guide the diagnosis of rheumatic fever, were not published until 1944 [S10,S11]. Although rheumatic fever typically presents with painful joints and carditis, these symptoms also occur in vitamin C deficiency. Notably, Glazebrook and Thomson observed reduced morbidity from streptococcal infections (Table S2) – the primary cause of rheumatic fever – raising the possibility that vitamin C might have influenced its occurrence. However, due to the lack of diagnostic details, it remains unclear how closely the reported rheumatic fever cases align with today’s definition of rheumatic fever. Despite this uncertainty, the reported difference between the low-dose (10-15 mg/day) and high-dose vitamin C groups remains valid, although the exact nature of the outcome is not well defined.

S10. Jones TD. The diagnosis of rheumatic fever. JAMA. 1944;126:481–484. https://doi.org/10.1001/jama.1944.02850430015005

S11. Gewitz MH, et al. Revision of the Jones Criteria for the diagnosis of acute rheumatic fever in the era of Doppler echocardiography. Circulation. 2015;131:1806-1818. https://doi.org/10.1161/cir.0000000000000205

Methods of the Glazebrook and Thomson (1942) trial are described in the report [24]. The structure of the paper is quite different from modern trial reports. Extracted here are the main descriptions:

> In a large training school under our observation there were some 1500 youths aged 15-20 years. For the most part they were drawn from the lower wage-earning classes, and a large proportion came from Scotland and the North Midlands, where economic conditions are probably below the average for the country. It is a reasonable assumption that the previous dietary of the recruits had been somewhat deficient in vitamin C judged by the standards already quoted (p.4).
>
> The food distribution [at the school] was badly managed. Electric ovens were used to reheat the food, and to keep it hot whilst awaiting distribution. Often 8 hr. elapsed between the time the food was cooked and its arrival on the dining tables. The minimum time that heat was applied to the food, including the original cooking and the subsequent reheating, was 2 hr (p.4).
>
> The total intake of vitamin C varied from about 10 to 15 mg per student per day (p.5).
>
> The calcium and vitamin B content of the dietary of the institution could perhaps be criticized, but the only outstanding deficiency, according to modern standards, was in vitamin C. As far as this one factor was concerned, the boys were almost certainly worse off, subsisting on the institution diet, than they would have been at home (p.17).
>
> Pure ascorbic acid powder was added to… the morning cocoa, and an evening glass of milk. The mixing was done in bulk in the kitchens before issue. The powder dissolved quickly and easily, and did not alter the appearance or taste of the vehicle (p.7).

Thus, the trial corresponds functionally to a placebo-controlled trial because the participants were unable to identify the treatment, although no inactive powder was added to the cocoa and milk of the control group.

> The establishment was divided into seven groups or divisions for administrative purposes. The youths of one division worked as a unit, and occupied certain tables in the dining hall. To some extent each division occupied particular dormitories but this separation was not absolute, and there was a fair amount of mixing of divisions in the sleeping quarters. Sleeping and feeding conditions were, of course, the same for all divisions (p.12).
>
> Careful records had been kept of the incidence of all infections for 1½ years before the observations described here were begun. In the preceding year there had been an epidemic of tonsillitis, which had affected all the divisions uniformly, so that they could not be regarded as separate units within the larger populations (p.12).

The above suggests that exposure to infectious agents was reasonably uniform over the study population.

> The observations were made by supplying vitamin C in the form of pure ascorbic acid to one or more divisions. This was considered to be the only practical method of carrying out the observations without introducing unnecessary complications. For example, it was not possible to choose boys at random as it would have been impossible to supply them with vitamin C-treated cocoa or milk in the dining room. With the method actually chosen, all that was necessary was to add vitamin C to the supplies of cocoa or milk serving the tables for the appropriate divisions (p.12).
>
> Moreover, all of the divisions had a population more or less the same as regards duration of stay in the establishment (‘institution age’). Infectious diseases were more common amongst those who had more recently joined the institution (p.12).
>
> When a youth fell ill he was admitted to Sick Quarters unless his complaint was very mild. In the latter case he was placed on the out-patients list and excused all duties except attendance at school instruction. Most of the cases of common cold and tonsillitis were admitted to Sick Quarters. In analysing the durations of illnesses, observations were restricted to the cases in the Sick Quarters. The number of days spent there was obviously a more reliable index of the duration of illness, since the

patient was under constant medical supervision. Frequently when a youth was discharged from the Sick Quarters he was put on the out-patients list, and this ‘convalescent period’ was neglected. The admission to and discharge from the hospital was not under our control (p.13).

The diet in the Sick Quarters was basically similar to that of the healthy boys. It was modified, of course, to suit the needs of the sick, but was prepared in the central kitchens and suffered an equally drastic loss of its vitamin C. When a student from the experimental division fell ill and was admitted to Sick Quarters, his dosage of ascorbic acid was continued there (p.14).

The period of treatment of cases of tonsillitis and common cold in the Sick Quarters was completely outside our control, and no biased attitudes influenced these durations from which we have drawn our conclusions (p.16).

[pneumonia… rheumatic fever] … These cases were-subjected to special investigations by us (X-rays, etc.) to establish certain criteria for the diagnosis. There was, however, in our opinion a relationship between these conditions (p.16).

It was not stated whether the diagnosis of pneumonia was carried out by the trial authors of the paper or by the physicians at the Sick Quarters. Although the method of diagnosing pneumonia was not described in detail in the paper, with the given descriptions and the severe pathological processes occurring in pneumonia it seems unlikely that administration of vitamin C in cocoa and milk could have led to detection bias.

21. Glazebrook AJ, Thomson S. The administration of vitamin C in a large institution and its effect on general health and resistance to infection. J Hyg (Lond). 1942;42:1-19.
https://doi.org/10.1017/s0022172400012596
https://pmc.ncbi.nlm.nih.gov/articles/PMC2199803

Related papers:
https://pmc.ncbi.nlm.nih.gov/articles/PMC2239225 [Description of the trial]
https://pmc.ncbi.nlm.nih.gov/articles/PMC2162337
https://pmc.ncbi.nlm.nih.gov/articles/PMC2164503
https://pmc.ncbi.nlm.nih.gov/articles/PMC2164703
https://pmc.ncbi.nlm.nih.gov/articles/PMC2210931
https://pmc.ncbi.nlm.nih.gov/articles/PMC2286225
https://doi.org/10.5281/zenodo.14609923

Linus Pauling’s analyses of the Glazebrook (1942) trial:
https://scarc.library.oregonstate.edu/coll/pauling/rnb/31/31-103.html
https://scarc.library.oregonstate.edu/coll/pauling/rnb/33/33-034.html
https://scarc.library.oregonstate.edu/coll/pauling/rnb/33/33-035.html

24. Hemilä H, Chalker E. Vitamin C for preventing and treating the common cold. Cochrane Database Syst Rev. 2013;2013:CD000980.
https://pmc.ncbi.nlm.nih.gov/articles/PMC8078152
https://hdl.handle.net/10138/225864
https://www.researchgate.net/publication/273209193
https://doi.org/10.1002/14651858.cd000980.pub4

**Ludvigsson (1977) Pilot [19]**	https://www.academia.edu/69567236 https://doi.org/10.3109/inf.1977.9.issue-2.07
Methods	Double-blind RCT. Regular supplementation trial. Duration 7 weeks in the spring of 1973. Pilot study to Ludvigsson (1977) Main, see below.
Participants	Swedish school children. 80 vitamin C (41 male, average age 9.61 yr); 78 placebo (42 male, average age 9.55 yr).
Intervention	1 g/d vitamin C.
Outcomes	Incidence, duration and severity of colds (Table V, p 95)
Baseline vit C intake	**190 mg/day** Placebo contained 30 mg/d vitamin C. Dietary intake ~160 mg/day.
Source of dose	Dietary intake estimated from published plasma vitamin C levels in [34].
Notes	Pilot study to Ludvigsson (1977) Main, see below

**Risk of bias**	**Authors' judgment**	**Support for judgment**
Random sequence generation (selection bias)	Low risk	"Every class was divided at random into two groups" (p.91).
Allocation concealment (selection bias)	Low risk	"carried out totally double blind" (p.92). At the end of the study "the code used [was] decoded" (p.92).
Baseline balance	Low risk	Table I shows balance for age and sex.
Blinding of participants and personnel (performance bias)	Low risk	"carried out totally double blind" (p.92). At the end of the study "the code used [was] decoded" (p.92).
Blinding of outcome assessment (detection bias)	Low risk	"carried out totally double blind" (p.92). At the end of the study "the code used [was] decoded" (p.92).
Incomplete outcome data (attrition bias)	Low risk	Of the 172 children who started in the pilot study 14 dropped out (p.93). The dropout rate due to suspected side effects was 1% (1/80) in the vitamin C group compared with 3% (2/78) in the placebo group.
Selective reporting (reporting bias)	Low risk	Incidence of colds, duration of colds and severity of colds reported. Several comparisons with P>0.05 were also reported.
Vitamin C and placebo indistinguishable?	Low risk	"fizzy tablet which contained 1000 mg vitamin C; in the other group the fizzy tablet looked and tasted the same" (p.91).

	**Placebo**	**Vitamin C**
Participants	78	80
"Upper respiratory tract infection" per episode, days	14.53	8.90
Difference		-39% **P = 0.0011** Table V [19]: t = 3.05

**Ludvigsson (1977)**	https://www.academia.edu/69567236
**Main [19]**	https://doi.org/10.3109/inf.1977.9.issue-2.07
Methods	Double-blind RCT. Regular supplementation trial. Duration 3 months in the Spring of 1973.
Participants	Swedish school children. 304 vitamin C (161 male, average age 9.31 years); 311 placebo (155 male, average age 9.31 years)
Intervention	1 g/d vitamin C.
Outcomes	Incidence, duration and severity of colds (Table V, p 95)
Baseline vit C intake	**170 mg/day**<br>Placebo contained 10 mg/d vitamin C.<br>Dietary intake ~160 mg/day.
Source of dose	Dietary intake estimated from published plasma vitamin C levels in [34].
Notes	

Risk of bias	Authors' judgment	Support for judgment
Random sequence generation (selection bias)	Low risk	"Every class was divided at random into two groups" (p.91).
Allocation concealment (selection bias)	Low risk	"carried out totally double blind" (p.92).<br>At the end of the study "the code used [was] decoded" (p.92).
Baseline balance	Low risk	Table I shows balance for age and sex.
Blinding of participants and personnel (performance bias)	Low risk	"carried out totally double blind" (p.92).<br>At the end of the study "the code used [was] decoded" (p.92).
Blinding of outcome assessment (detection bias)	Low risk	"carried out totally double blind" (p.92).<br>At the end of the study "the code used [was] decoded" (p.92).
Incomplete outcome data (attrition bias)	Low risk	Of the 642 children who started in the main study 27 dropped out (p.93). The dropout rate due to suspected side effects was 0.3% (1/304) in the vitamin C group compared with 0.3% (1/311) in the placebo group
Selective reporting (reporting bias)	Low risk	Incidence of colds, duration of colds and severity of colds reported. Several comparisons with P>0.05 were also reported.
Vitamin C and placebo indistinguishable?	Low risk	"fizzy tablet which contained 1000 mg vitamin C; in the other group the fizzy tablet looked and tasted the same" (p.91).

	**Placebo**	**Vitamin C**
Participants	311	304
"Absence from school because of upper respiratory tract infection"		
per episode, days	3.22	2.77
Difference		-14%<br>**P = 0.008**<br>Table V [19]: t = 2.42

**Miller (1977) [18]**	https://doi.org/10.1001/jama.1977.03270300052006<br>https://pubmed.ncbi.nlm.nih.gov/318715
Methods	Double-blind RCT. Regular supplementation trial. Duration 5 months beginning in November 1974.
Participants	US school children, ranging from 6 to 15 yr. 44 twin pairs (18 boy pairs)<br><br>Identical twins.<br>The twin pairs were separated by body weight into three dosage groups receiving 0.5 g, 0.75 g or 1 g ascorbic acid daily (p.248).
Intervention	0.5–1 g/d vitamin C.
Outcomes	Incidence, duration and severity of colds (Table 3, p 259).<br>Identifying the twin who received the vitamin C supplement from personal observations by the mother (p.250).
Baseline vit C intake	**530 mg/day**<br>Placebo contained 50 mg/d vitamin C.<br>"All twins received a multiple vitamin preparation that contained 50 mg of vitamin C in order to ensure that any observed treatment effect could reasonably be attributed to pharmacologic doses of the vitamin rather than to dietary deficiency" (p.248-9).<br><br>Before the trial the placebo group excreted on average 314 mg/day vitamin C in urine.
Source of dose	Dietary intake estimated from published plasma vitamin C levels in [34].
Notes	This study is shown in Tables S1 and S2 since it is a vitamin C and common cold trial. However, although the small P-value indicates observable effects of vitamin C administration, the outcome in Table S1 is not a direct respiratory infection outcome and therefore the trial is not included in Figure 1.

**Risk of bias**	**Authors' judgment**	**Support for judgment**
Random sequence generation (selection bias)	Low risk	"Within a twin pair, the assignment to the treatment group was random" (p.248).
Allocation concealment (selection bias)	Low risk	"double-blind"; "The code was not broken until after the analysis of symptom data had been completed" (p.248).
Baseline balance	Low risk	Twins.
Blinding of participants and personnel (performance bias)	Low risk	"double-blind"; "The code was not broken until after the analysis of symptom data had been completed" (p.248).
Blinding of outcome assessment (detection bias)	Low risk	"double-blind"; "The code was not broken until after the analysis of symptom data had been completed" (p.248).
Incomplete outcome data (attrition bias)	Low risk	1 pair of boy twins was omitted from the analysis because of incomplete data (p.249).
Selective reporting (reporting bias)	Low risk	Incidence of colds, duration of colds and severity of colds were reported. Several comparisons with P>0.05 were reported.
Vitamin C and placebo indistinguishable?	Low risk	"The capsules contained 250 mg vitamin C or starch" (p.248)<br>"Four mothers acknowledged tasting the contents of the capsules… cannot exclude the possibility… that they recognized the vitamin C by taste" (p.251).

Inferring vitamin C by mothers of the twins:

"*As a final approach to the detection of subtle treatment effects on general well-being that might not have been measured by any of our tests, each mother was asked to guess, while she and the investigator were still blinded, which twin had received the vitamin. Twenty-three of the 44 mothers could not tell any difference between the treated and control twins. However, among the 21 mothers who felt there was a detectable effect of treatment, 17 correctly identified the twin who had received the vitamin (P < 0.05). Among the mothers of the young twins in whom the objective evidence for a treatment effect was the greatest, eight of nine guessed correctly (P < 0.02)*" (p.250.)

For the 4/17 split, the binomial distribution gives P(1-tail) = 0.004:

```
> binom.test(17, 21, p = 0.5, alternative = "greater")
      Exact binomial test
data:  17 and 21
number of successes = 17, number of trials = 21, p-value = 0.00359
alternative hypothesis: true probability of success is greater than 0.5
```

Of the 44 mothers, 4 mothers guessed the treatment incorrectly and that needs to be subtracted from the 21 mothers who gave the correct answer (compare analysis in [S1, p.26, Table 11]. Thus, we conclude that 39% (17/44) of the mothers could infer the administration of vitamin C correctly.

S1. Hemilä H. Do vitamins C and E affect respiratory infections?
University of Helsinki, Helsinki, Finland 2006.
https://hdl.handle.net/10138/20335
https://doi.org/10.5281/zenodo.6395595

**Peters (1993) [29]**	https://doi.org/10.1093/ajcn/57.2.170<br>Discussion:<br>https://doi.org/10.1093/ajcn/59.1.132<br>https://doi.org/10.1093/ajcn/59.1.132a
Methods	Double-blind RCT. Regular supplementation trial. Duration 3 weeks before the 90 km Comrades Marathon 1990.
Participants	South Africa. 84 ultramarathon runners (82 male). 43 vitamin C; 41 placebo. Five were <25 yr, 57 were 25-40 yr, 22 were > 40 yr (p.172).
Intervention	0.6 g/d vitamin C for 3 weeks before the race, but not after the race
Outcomes	Outcome assessment continued for 2 weeks after the race.<br>Incidence and duration of colds (Table 3, p 173).
Baseline vit C intake	**494 mg/day**<br>*Placebo group:*<br>Supplement on own initiative 209 mg/day (included in the 494 mg/d below).<br>Total baseline vitamin C: 494 mg/day<br><br>*Vitamin C group:*<br>Supplement on own initiative 227 mg/day (included in the 547 mg/d below).<br>Total baseline vitamin C: 547 mg/day
Source of dose	Baseline daily vitamin C intake is reported in Table 1.<br>Mean supplement on own initiative is reported in Table 2, but there is no separate report of dietary vitamin C intake.
Notes	A parallel trial examined sedentary people with a similar design.

**Risk of bias**	**Authors' judgment**	**Support for judgment**
Random sequence generation (selection bias)	Low risk	"were randomly divided into" (p.170).
Allocation concealment (selection bias)	Low risk	"double-blind" (p.170).
Baseline balance	Unclear risk	Baseline balance not demonstrated.
Blinding of participants and personnel (performance bias)	Low risk	"double-blind" (p.170).
Blinding of outcome assessment (detection bias)	Low risk	"double-blind" (p.170).
Incomplete outcome data (attrition bias)	Low risk	"8 runners failed to comply ith all requirements of the protocol and were excluded" (p.171).
Selective reporting (reporting bias)	Low risk	Incidence and duration of colds reported. A parallel trial with sedentary participants was similarly reported. Comparisons with P>0.05 were reported.
Vitamin C and placebo indistinguishable?	Low risk	"identical looking and tasting placebo containing citric acid" (p.170)

	**Placebo**	**Vitamin C**
Participants	41	43
Number of colds	28	14
Difference		-53.0%<br>**P = 0.0006**

Calculation of the P-value for the difference in incidence:

```
> riskratio.wald(Peters)
$data
          Outcome
Predictor  Disease1 Disease2 Total
  Exposed1       14       29    43
  Exposed2       28       13    41
  Total          42       42    84

$measure
          risk ratio with 95% C.I.
Predictor  estimate     lower     upper
  Exposed1 1.000000        NA        NA
  Exposed2 0.470143 0.2866128 0.7711951

$p.value
          two-sided
Predictor   midp.exact fisher.exact  chi.square
  Exposed1          NA           NA          NA
  Exposed2 0.001233025  0.002055608 0.001059629


> (P_1tail=0.00123/2)
[1] 0.000615
```

**Pitt (1979) [21]**	https://www.academia.edu/78271637<br>https://doi.org/10.1001/jama.1979.03290350028016
Methods	Double-blind RCT. Regular supplementation trial. Duration 8 weeks.
Participants	USA male marine recruits. 331 vitamin C (mean age 18.5 yr); 343 placebo (mean age 18.5 yr).
Intervention	2 g/d vitamin C. "Pill taking was supervised and observed by the drill instructors in each platoon" (p.908).
Outcomes	Incidence, duration and severity of colds (Table 2, p 910).<br>"Pneumonia developed in eight recruits… Each of these eight recruits had typical roentgenographic and physical signs of pneumonia…" (p.910).
Baseline vit C intake	**150 mg/day**
Source of dose	Dietary intake estimated from published whole blood vitamin C levels in [34]. The relation of vitamin C level in plasma and in whole blood has been examined and they are close enough [S12-S14] for the purpose of our analysis. In addition, red blood cells contain somewhat lower level of vitamin C than plasma [S15,S16], which would lead to bias downwards rather than upwards when comparing whole blood and plasma.
Notes	The severity of colds was classified on a numerical rating from 1 to 4. Since the minimum of their scale was 1, for our analysis we rescaled the severity levels for the range from 0 to 3.<br><br>In Table 2 Pitt reported:<br>Placebo 1.97 severity score<br>Vitamin C 1.87 severity score<br><br>In our rescaling we used the following values:<br>Placebo 0.97 severity score corrected<br>Vitamin C 0.87 severity score corrected<br>Decrease -10.3%<br>P 0.012<br>[Table 2 [21]: $Chi^2(15\ df) = 27.8$, which corresponds to P(2-tail) = 0.023]

Risk of bias	Authors' judgment	Support for judgment
Random sequence generation (selection bias)	Low risk	"were assigned randomly to either the vitamin C or placebo group… from a list of consecutive numbers randomized in pairs. Randomization was carried out by individual recruits within each platoon" (p.908).
Allocation concealment (selection bias)	Low risk	"Neither the recruits or drill instructors nor the physicians and corpsmen who treated the recruits were aware of which pill any individual recruit was taking" (p.908).
Baseline balance	Low risk	Table 1 shows balance for age, race, previous medical history, previous cold history, work days lost per year (p.909).
Blinding of participants and personnel (performance bias)	Low risk	"Neither the recruits or drill instructors nor the physicians and corpsmen who treated the recruits were aware of which pill any individual recruit was taking" (p.908).
Blinding of outcome assessment (detection bias)	Low risk	"Neither the recruits or drill instructors nor the physicians and corpsmen who treated the recruits were aware of which pill any individual recruit was taking" (p.908).
Incomplete outcome data (attrition bias)	Low risk	Of the 862 recruits who began taking the pills, 64 (34 vitamin C, 30 placebo) were removed from their platoons. An additional 123 recruits (64 vitamin C, 59 placebo<br>were excluded from the final analysis because they did not continue to take their pills for the full study period (p.909)<br>The dropout rates were 22.8%(98/429) in the vitamin C group, compared with 20.6% (89/432) in the placebo group.
Selective reporting (reporting bias)	Low risk	Incidence, duration and severity of colds, and the incidence of pneumonia were reported. Comparisons with P>0.05 were reported.

Vitamin C and placebo indistinguishable?	Low risk	Sinusitis, otitis media, tonsillitis, streptococcal pharyngitis, and bronchitis were not reported because incidence was too low. "The placebo tablets were formulated from citric acid and were indistinguishable in appearance and taste from the vitamin C tablets" (p.908).

	**Placebo**	**Vitamin C**
Participants	343	331

The severity of colds was classified on a numerical rating from 1 to 4. Since the minimum of their scale was 1, for our analysis we rescaled the severity levels for the range from 0 to 3 [53].

Severity of colds	0.97	0.87
Difference		-10.3%
		**P = 0.012**
		Table 2 [21]: Chi^2(15 df) = 27.8
		which corresponds to P(2-tail) = 0.023

Cases of pneumonia (p.910):
"*Pneumonia developed in eight recruits, and only one of these recruits was from the vitamin C group (P<.04). Each of these eight recruits had typical roentgenographic and physical signs of pneumonitis, although five recruits were febrile, and only four recruits had elevated WBC counts. Pneumococci were isolated from the sputum in three recruits and seen intracellularly on Gram's stain in two other recruits. Two of these recruits also had fourfold increases in parainfluenza titers*".

Cases of pneumonia	7	1
Difference		-87%
		**P = 0.021**

```
riskratio.small(Pitt)
$data
          Outcome
Predictor  Disease1 Disease2 Total
  Exposed1      336        7   343
  Exposed2      330        1   331
  Total         666        8   674

$measure
          risk ratio with 95% C.I.
Predictor   estimate      lower    upper
  Exposed1 1.0000000         NA       NA
  Exposed2 0.1299094 0.01607068 1.050139

$p.value
          two-sided
Predictor  midp.exact fisher.exact chi.square
  Exposed1         NA           NA         NA
  Exposed2 0.04268428   0.06903668 0.03718801

> (P_1tail=0.04268/2)
[1] 0.02134
```


S12. https://doi.org/10.1172/jci101004 Heinemann J Clin Invest 1938
S13. https://doi.org/10.1172/jci101637 Lubschez J Clin Invest 1945
S14. https://doi.org/10.1172/jci101816 Roe et al. J Clin Invest 1947
S15. https://doi.org/10.1016/j.ab.2012.04.014 Li et. al. Anal Biochem 2012
S16. https://doi.org/10.3390/nu12020418 Pullar et al. Nutrients 2020

**Sabiston (1974) [23]**	https://doi.org/10.5281/zenodo.7303680<br>https://pubs.drdc-rddc.gc.ca/BASIS/pcandid/www/engpub/DDW?W%3DSYSNUM=23882&r=0
Methods	Double-blind RCT. Regular supplementation trial. Duration 2 to 3 weeks.
Participants	Canadian male military recruits during subarctic winter exercises. 56 vitamin C (mean age 25.3, range 17 to 40); 56 placebo (mean age 25.4, range 17 to 47).
Intervention	1 g/d vitamin C.
Outcomes	Incidence, duration and severity of colds (Table 4, p 5; Table 6, p 6).
Baseline vit C intake	**39 mg/day**<br>We use 0.039 g/day as an estimated baseline vitamin C intake in the Sabiston (1974) trial, though according to the text below, this is likely to be more than many men were receiving.<br>"One further point with reference to Table 8 is the rather surprising number of men who demonstrated whole-blood ascorbate levels lower than 0.50 mg%. This value is generally taken to indicate the threshold of a possible sub-clinical scorbutic condition" (p.7).
Source of dose	"it was determined that the RP-4 rations (1970-71) on which the men were living, apparently provided a maximum of 37–41 mg Vitamin C per day in a single fruit-drink mix. As previous observations suggested that the fruit-drink mix was an unpopular item in the rations and tended to be discarded, it appeared that the individual intake of Vitamin C could be below the recommended daily allowance" (p.4).
Notes	Personal communication from Manny Radomski (email 12 September 2009): "Tent group commanders [who were responsible for distributing the pills and recording the distribution] did not know what was in the vials… We [the authors] collected the data by symptoms on T-scan cards. We did not 'break the code' until after all cards had been assessed."<br><br>Funding: Canadian Army

**Risk of bias**	**Authors' judgment**	**Support for judgment**
Random sequence generation (selection bias)	Low risk	"Men in each tent were assigned randomly… " (p.4)<br><br>"we did assign people randomly. We had the names of people beforehand but we assigned them randomly and we provided their names on the pill vials. The Tent Group Commander was responsible for distributing the pills and recording the distribution. He did NOT know what was in the vials… While we pre-assigned Vit C and Placebo randomly,we did not break the code until after the trial. Two labelled vials were provided to the Tent Group Commanders but the Tent Group Commanders did NOT know what was in the vials" (email Radomski 12 September 2009).
Allocation concealment (selection bias)	Low risk	"we did assign people randomly. We had the names of people beforehand but we assigned them randomly and we provided their names on the pill vials. The Tent Group Commander was responsible for distributing the pills and recording the distribution. He did NOT know what was in the vials… While we pre-assigned Vit C and Placebo randomly, we did not break the code until after the trial. Two labelled vials were provided to the Tent Group Commanders but the Tent Group Commanders did NOT know what was in the vials" (email Radomski 12 September 2009).
Baseline balance	Low risk	Table 2 shows that age and common cold history were balanced
Blinding of participants and personnel (performance bias)	Low risk	"The Tent Group Commander was responsible for distributing the pills and recording the distribution. He did NOT know what was in the vials… While we pre-assigned Vit C and Placebo randomly, we did not break the code until after the trial. Two labelled vials were

		provided to the Tent Group Commanders but the Tent Group Commanders did NOT know what was in the vials" (email Radomski 12 September 2009).
Blinding of outcome assessment (detection bias)	Low risk	"we did assign people randomly. We had the names of people beforehand but we assigned them randomly and we provided their names on the pill vials. The Tent Group Commander was responsible for distributing the pills and recording the distribution. He did NOT know what was in the vials… While we pre-assigned Vit C and Placebo randomly, we did not break the code until after the trial. Two labelled vials were provided to the Tent Group Commanders but the Tent Group Commanders did NOT know what was in the vials" (email Radomski 12 September 2009).
Incomplete outcome data (attrition bias)	Low risk	Short study in military conditions.
Selective reporting (reporting bias)	Low risk	Incidence, duration and severity of colds reported. Comparisons with P>0.05 were reported.
Vitamin C and placebo indistinguishable?	Low risk	"Vitamin C and placebo were in identical capsules, so taste did not enter into the equation… In our pre-briefing to the troops, we believe that we told the troops that they would all be getting vitamin C but at different doses" (email Radomski 12 September 2009).

	**Placebo**	**Vitamin C**
Participants	56	56
Number of colds	14	6
Difference		-59.3%<br>**P = 0.027**
Duration of severe colds, days	2.40	0.80<br>-66.7%<br>**P=0.010**

```
riskratio.small(Sabiston)
$data
          Outcome
Predictor  Disease1 Disease2 Total
  Exposed1       42       14    56
  Exposed2       50        6    56
  Total          92       20   112

$measure
          risk ratio with 95% C.I.
Predictor   estimate     lower     upper
  Exposed1 1.0000000        NA        NA
  Exposed2 0.4071429 0.1685849 0.9832748

$p.value
          two-sided
Predictor  midp.exact fisher.exact chi.square
  Exposed1         NA           NA         NA
  Exposed2 0.05354471   0.08242985 0.04841124

> (P_1tail=0.0535/2)
[1] 0.02675
```

The P-value for the Sabiston (1974) trial is calculated with the Ratio of Means approach as described by Friedrich [S5], see section “Statistical issues”.

```
ROM
                      Study  Ne     Me    Se  Nc     Mc   Sc
1                 Friedrich   9 213.00 67.00  10 177.00 40.0
2             Sabiston 1974   6   0.80  0.80  14   2.40  2.1
> ROM$LnROM   = log(ROM$Me/ROM$Mc)
> ROM$SDe     = (ROM$Se/ROM$Me)**2/ROM$Ne
> ROM$SDc     = (ROM$Sc/ROM$Mc)**2/ROM$Nc
> ROM$SElnROM = sqrt(ROM$SDe + ROM$SDc)
> ROM$z       = ROM$LnROM/ROM$SElnROM
> ROM$p       = pnorm(ROM$z)
> ROM
                      Study  Ne     Me    Se  Nc     Mc   Sc  LnROM    SDe     SDc SElnROM     z        p
1                 Friedrich   9 213.00 67.00  10 177.00 40.0  0.185 0.0110 0.00511   0.127  1.46 9.28e-01
2             Sabiston 1974   6   0.80  0.80  14   2.40  2.1 -1.099 0.1667 0.05469   0.470 -2.34 9.77e-03
```

S5. Friedrich JO, Adhikari NK, Beyene J. Ratio of means for analyzing continuous outcomes in meta-analysis performed as well as mean difference methods. J Clin Epidemiol. 2011;64:556–64. https://doi.org/10.1016/j.jclinepi.2010.09.016
